# HDAC3 inhibition mitigates acute kidney injury by alleviating RIPK1-mediated programmed necrosis

**DOI:** 10.3389/fphar.2025.1546950

**Published:** 2025-04-25

**Authors:** Manman Xie, Rui Hou, Runrun Shan, Xinyu Cheng, Pengcheng Wu, Xiufeng Luo, Yangyang Wei, Li Gao, Xiaoying Liu, Qi Chen

**Affiliations:** ^1^ School of Life Sciences, Anhui Medical University, Hefei, 230032, China; ^2^ School of Pharmacy, Anhui Medical University, Hefei, 230032, China; ^3^ State Key Laboratory of Pharmaceutical Biotechnology, College of Life Sciences, Nanjing University, Nanjing, 210023, China; ^4^ The Armed Police Corps Hospital of Anhui, Hefei, China; ^5^ Department of Nephropathy, The First Affiliated Hospital of Anhui Medical University, Hefei, 230022, China; ^6^ Henan International Joint Laboratory of Non-coding RNA and Metabolism in Cancer, Henan Provincial Key Laboratory of Long Non-coding RNA and Cancer Metabolism, Translational Research Institute of Henan Provincial People’s Hospital and People’s Hospital of Zhengzhou University, Zhengzhou, Henan, China

**Keywords:** acute kidney injury, necroptosis, RGFP966, inflammation, HDAC3 abbreviations Cr: creatinine, RG: RGFP966, IF: immunofluorescence, HK2: human kidney tubular epithelial cells

## Abstract

Acute kidney injury (AKI) refers to clinical syndromes culminating in rapidly reduced renal function associated with inflammation and the demise of renal tubular epithelial cells. Current research aims to develop strategies which prevent tubular cell death. Here, based on the involvement of histone deacetylases (HDACs) in renal physiology and their established role in renal fibrosis, we investigated the mechanistic contributions of HDACs using a mouse model together with *in vitro* studies employing human renal epithelial cells. We found HDAC3 expression was upregulated in mouse renal tubules after ischemia/reperfusion and cisplatin treatment. Instructively, treatment with the HDAC3 selective inhibitor RGFP966 exerted potent protective effects, attenuates acute kidney injury in both *in vivo* and *in vitro* models. Moreover, RGFP966 was found to reduce inflammation and injury caused by cisplatin and hypoxia-reoxygenation in HK2 cells with transcriptome sequencing revealing that RGFP966 significantly inhibited the upregulation of the necroptosis initiator, RIPK1. Cellular thermal displacement assay and molecular docking demonstrated the physical binding of RGFP966 to HDCA3. In addition, RIPK1 knockdown cell assay signified that RGFP966 targeted RIPK1 and inhibited RIPK1 kinase activity. In summary, these findings established the efficacy of the HDAC3 inhibitor RGFP966 in treating AKI.

## 1 Introduction

Acute kidney injury (AKI) is marked by a rapid decline in renal function and is associated with a wide array of underlying causes. This condition significantly affects both immediate morbidity and mortality, while also influencing patients’ long-term prognosis (Pickkers et al., 2021; Ronco et al., 2019). The global incidence of AKI-related mortality surpasses that of diabetes mellitus, heart disease, and many common cancer types, remaining alarmingly high over the past 5 decades. This persistently elevated mortality imposes a significant socioeconomic burden, straining healthcare systems and affecting broader societal wellbeing (Lewington et al., 2013; Hoste et al., 2018). Despite advancements in medical care, the mortality rate for patients requiring kidney transplantation remains above 50%, largely because specific medications to prevent or treat AKI are not yet available. As a result, supportive care and dialysis remain the primary treatment options (Zuk and Bonventre, 2015; Levey and James, 2018; Kellum et al., 2021).

AKI is caused by various pathophysiologic factors, including inflammatory reactions and the necrotic demise of proximal renal epithelial cells in the tubule (Liu et al., 2020; Gao et al., 2018). Regarding the latter, a combination of programmed cell death pathways including apoptosis, necroptosis, ferroptosis, pyroptosis, and autophagy-associated death contribute to the rapid decline of renal function. Among these pathways, apoptosis and necroptosis have been more often associated with severe or prolonged kidney injury (Frank and Vince, 2019a). In any case, developing effective strategies at blocking the induction of cell death pathways has promising therapeutic potential to modulating cell death and reducing AKI in the clinic.

Necroptosis is initiated by the formation of “necrosomes”, the oligomeric complexes comprising receptor-interacting kinase 1 (RIPK1) and RIPK3, with RIPK1 acting as a key initiator (Grootjans et al., 2017; Zhao et al., 2020).

The interplay of histone deacetylases (HDACs) with histone acetyltransferases (HAT) serves to regulate the dynamic post-translational modification of histones by acetylation (Li and Seto, 2016; Cheshmazar et al., 2022). Previous literature has shown that HDACs fulfil critical functions in renal physiology and fibrosis (Fontecha-Barriuso et al., 2018; Hyndman, 2020; Hadden and Advani, 2018). For instance, HDAC1/HDAC2 have been reported to promote renal fibroblast proliferation in association with STAT3 activation (Tang et al., 2018). Notably, the application of small molecule HDAC inhibitors has been shown to effectively modulate a range of biological functions, including cell cycle regulation, proliferation, apoptosis, along with immune system control *in vitro* and *in vivo* (Brilli et al., 2013). For example, the HDAC6 inhibitor Tubastatin A was reported to increase autophagy and reduce renal injury (Zhang et al., 2023), and the HDAC9-selective inhibitor TMP195 was shown to attenuate renal fibrosis in the unilateral ureteral obstruction (UUO) mouse model (Amin et al., 2019).

However, the role of HDAC3 inhibitors in AKI is yet to be determined. Therefore, in this study, the expression and localization of HDAC3 were explored in an AKI model. The findings indicated that the HDAC3 inhibitor (2E)-N-(2-amino-4-fluorophenyl)-3-[(2E)-1-(3-phenyl-2-propen-1-yl)-1H-pyrazol -4-yl]-2-propenamide (RGFP966) alleviated injury and inflammation induced by cisplatin and hypoxia-reoxygenation (H/R) in HK2 cells. Moreover, the preventive and therapeutic impact of RGFP966 in cisplatin- and ischemia/reperfusion (I/R)-induced AKI mouse models were examined using different protocols. The molecular mechanisms of RGFP966 in its effects were also investigated through RNA sequencing (RNA-seq).

## 2 Materials and methods

### 2.1 Reagents and antibodies

The specific HDAC3 inhibitor, RGFP966, of 99.77% purity and cisplatin were purchased from TargetMol and MedChemExpress (MCE), respectively. Antibodies against KIM-1 (T58586S; Abmart), HDAC3 (10,255-1- AP), p65 (WL01273b, Wanleibio), p-p65 (WL012169, Wanleibio), RIPK3 (ER190127; HUABIO), p-RIPK3 (phosphor S232, ab195117, Abcam), RIPK1 (ET1701-79; HUABIO), p-RIPK1 (phosphor S166, AF2398, HUABIO), p-MLKL (ET1705-51, HUABIO) and β-actin (GB12044; Servicebio) were sourced as indicated. Secondary IRDye 800-conjugated antibodies were purchased from LI-COR Biosciences (No. C60113-02).

### 2.2 Establishment of AKI mouse models

Male C57BL/6J mice (weight: 20–23 g) were obtained from the Hangzhou Ziyuan Laboratory Animal Technology Company. All experiments were performed at Anhui Medical University under approvals granted by the Animal Experimentation Ethics Committee of Anhui Medical University (LLSC20241408). Animal care strictly followed the 3R principle according to humane care guidelines. After acclimatization, mice were randomly divided into groups of six for each experimental group in the cisplatin and I/R models. For the cisplatin model, mice received intraperitoneal injection (i.p.) of 20 mg/kg cisplatin with the result. Alternatively, I/R surgery was performed under anesthesia with mouse body temperature maintained at 36.5°C. Briefly, bilateral renal pedicles were clipped for 40 min using microaneurysm clamps. After clamp withdrawal, kidneys were reperfused for 24 h before the mice were sacrificed. Control group animals were subjected to identical procedures except for the renal pedicle clamping. Interventions involved normal saline or RGFP966 (5, 10, and 20 mg/kg body weight i.p.) 12 h before cisplatin treatment, repeated once a day for three consecutive days or 20 mg/kg RGFP966 12 h before I/R. Pretreatment blood and kidney tissue samples were acquired under isoflurane anesthesia or after sacrifice to measure indicators of kidney injury and inflammation. FFPE kidney tissues were sectioned at 4 μm thickness and sections stained using the periodic acid-Schiff (PAS) staining kit (C0142S, Beyotime Biotechnology, Jiangsu, China).

### 2.3 Molecular docking

In silico docking analysis was used to model binding between RGFP966 and HDAC3 (Shanghai Tao Technology Biological Co., LTD.) using Discovery Studio 2017 R2 (DS, BIOVIA Software, Inc., San Diego, CA, United States) against the 3D model of HDAC3 (ID:4A69) obtained from the Protein Data Bank (PDB).

### 2.4 Cell culture and treatment

The immortalized human kidney cell line derived from normal proximal tubule epithelial cells (HK2) was purchased from Procell (Wuhan, China) and cultured in a DME/F-12 (1:1) medium containing 5% fetal bovine serum (FBS) and 5% CO_2_ at 37°C. The cells were pretreated with RGFP966 (2, 4, and 8 μM) and then stimulated with cell starvation for 12 h when cell confluence reached 50%. Next, the cells were co-incubated with or without cisplatin (20 μM) for additional 24 h. The cells were incubated in a thermostat incubator (37°C, 5% CO_2_) and a low-glucose solution containing 0.5% FBS at 37°C for 12 h to establish H/R injury model *in vitro.* The cells were then placed back to the regular environment for 6 h to allow reoxygenation. The H/R damage induction procedure was performed thrice, and the cells were harvested for analysis.

### 2.5 Cellular thermal shift assay

HK2 cells cultured in a 6-well plate were treated without or with RGFP966 (8 μM) for 2 h before harvesting the cells by trypsin digestion and transferring cell suspensions in culture medium to 1.5 mL centrifuge tubes. Tubes were then heated at either 42°C, 47°C, 52°C, 57°C, 62°C for 8–10 min before centrifugation at 800 rpm to collect the cells followed by Western blot analysis.

### 2.6 Western blotting

Proteins from tissues or cells were extracted with Frigid radioimmunoprecipitation assay buffer (Beyotime Biotechnology, Jiangsu, China) supplemented with phenylmethylsulfonyl fluoride, electrophoresed on 10% SDS-PAGE and transferred to nitrocellulose membranes. The membranes were blocked with 5% skim milk and washed 3–4 times with Tris-buffered saline containing 0.1% Tween 20. Subsequently, the membranes were incubated with specific primary antibodies overnight at 4°C. The membrane was washed 3–4 times the next day and incubated with a corresponding IRDye 800-coupled secondary antibodies for 2 h at room temperature. Epifluorescence images of the membranes were developed using the LiCor/Odyssey system (LI-COR Biosciences, Lincoln, NE).

### 2.7 RNA extraction and real-time quantitative PCR

Total RNA was extracted from tissues or cells using the AG RNAex Pro RNA Extraction Reagent (Accurate Biology, Hangzhou, China) as per the manufacturer’s recommendations. After measuring RNA concentrations using a NanoDrop 2000 Spectrophotometer (Thermo Scientific), cDNA was obtained from total RNA using the RealMasterMix (TOYOBO, Japan). Quantitative real-time PCR (qPCR) was performed using the target primers on a Bio-Rad (USA) CFX96 real-time instrument with data normalization against β-actin.

### 2.8 Determination of serum creatinine (Cr), aspartate aminotransferase (AST), blood urea nitrogen (BUN) and alanine aminotransferase (ALT) levels

Blood serum levels of Cr, AST, BUN and ALT were assessed using assay kits procured from Nanjing Jianjian Bioengineering Institute according to the manufacturer’s instructions.

### 2.9 Cell viability assay

HK2 cells seeded into 96-well plates with medium containing 5% FBS DME/F-12 (1:1) were cultured for 24 h before treatment with 0–128 μM RGFP966 for 12 h, followed by treatment without or with cisplatin (10 μM) for additional 24 h. After addition of 10 μL CCK-8 reagent/well and further incubation for 1–4 h, absorbance measurements were undertaken at 450 nm using a Multiskan FC plate reader (Thermo) with cell viability calculated as normalized data relative to control wells.

### 2.10 Tissue histology and immunofluorescence staining

Sections of formalin-fixed paraffin embedded kidney tissues were baked in a 65°C oven for 2 h, followed by sequential deparaffinization in xylene and rehydration in graded alcohols and water before staining using standard histology procedures or alternatively immunofluorescence staining against the indicated proteins. For the latter, heat-based (microwave) antigen retrieval was first performed using ethylene diamine tetraacetic acid (EDTA) solution before blocking with BSA solution for 30 min, after which the sections were incubated with primary antibodies at 4°C overnight. The next day, sections were washed three times with TBST for 10 min, further incubated with fluorophore-labelled secondary antibodies for 2 h at room temperature and then washed as above. Images were acquired using epifluorescence microscopy (Leica Microsystems GmbH., Wetzlar, Germany).

### 2.11 Immunofluorescent staining

HK2 cells cultured in eight-chamber glass slides were fixed in cold acetone. After blocking, the cells were then incubated with 1:200 diluted primary antibodies against KIM-1 or TNF-α overnight at 4°C. Primary antibodies were decorated with cow anti-rabbit IgG-rhodamine conjugate (Bioss Antibody Inc.) for 2 h at room temperature. After washing the cells three times with PBS, 4′,6-Diamidino-2-phenylindole (DAPI) was used as a nuclear counterstain. Images were acquired using a Leica epifluorescence microscopy as above.

### 2.12 Transcriptome sequencing (RNA-seq)

Total RNA was extracted from cells cultured in 6-well plates using TRIzol reagent with samples sent to BGI (BGI Genomics, Shengzhen, China) for sequencing and analysis with bioinformatics performed to determine differentially expressed genes (DEGs) and pathway enrichment.

### 2.13 Transmission electron microscopy (TEM)

HK2 cells fixed with 2.5% glutaraldehyde for 4–12 h were rinsed for 10 min in 0.1 M phosphate buffer at room temperature for three times. Next, dehydration was carried out using an increasing ethanol concentration series, followed by embedding in LR White resin (London Resin Company, Reading, UK). Samples were observed using TEM (H-7700, Tokyo, Japan).

### 2.14 SiRNA knockdown

HK2 cells were transfected with RIPK1 targeting siRNAs (5′-GCA​AAG​ACC​UUA​CGA​GAA​UUU​TT-3′) using Lipofectamine 2000 according to the manufacturers’ instructions. The siRNA-cationic liposome complexes prepared in Opti-MEM were incubated with the target cells for 6 h, before replacing the transfection reagents with fresh DMEM-F12 media without antibiotics. Afterwards, the cells were harvested for qPCR assays at 24 h or Western blotting assays at 48 h.

### 2.15 Statistical analyses

GraphPad Prism 8 (GraphPad, La Jolla, CA, U.S.A.) was used for data analyses. *P < 0.05; **P < 0.01; ***P < 0.001; ^#^P < 0.05; ^##^P < 0.01; ^###^P < 0.001. Data were expressed as either mean ± standard error (SEM) for 6-8 mice or 3-4 independent experiments.

## 3 Results

### 3.1 HDAC3 expression is elevated in both *in vivo* and *in vitro* models of acute kidney injury

**FIGURE 1:**
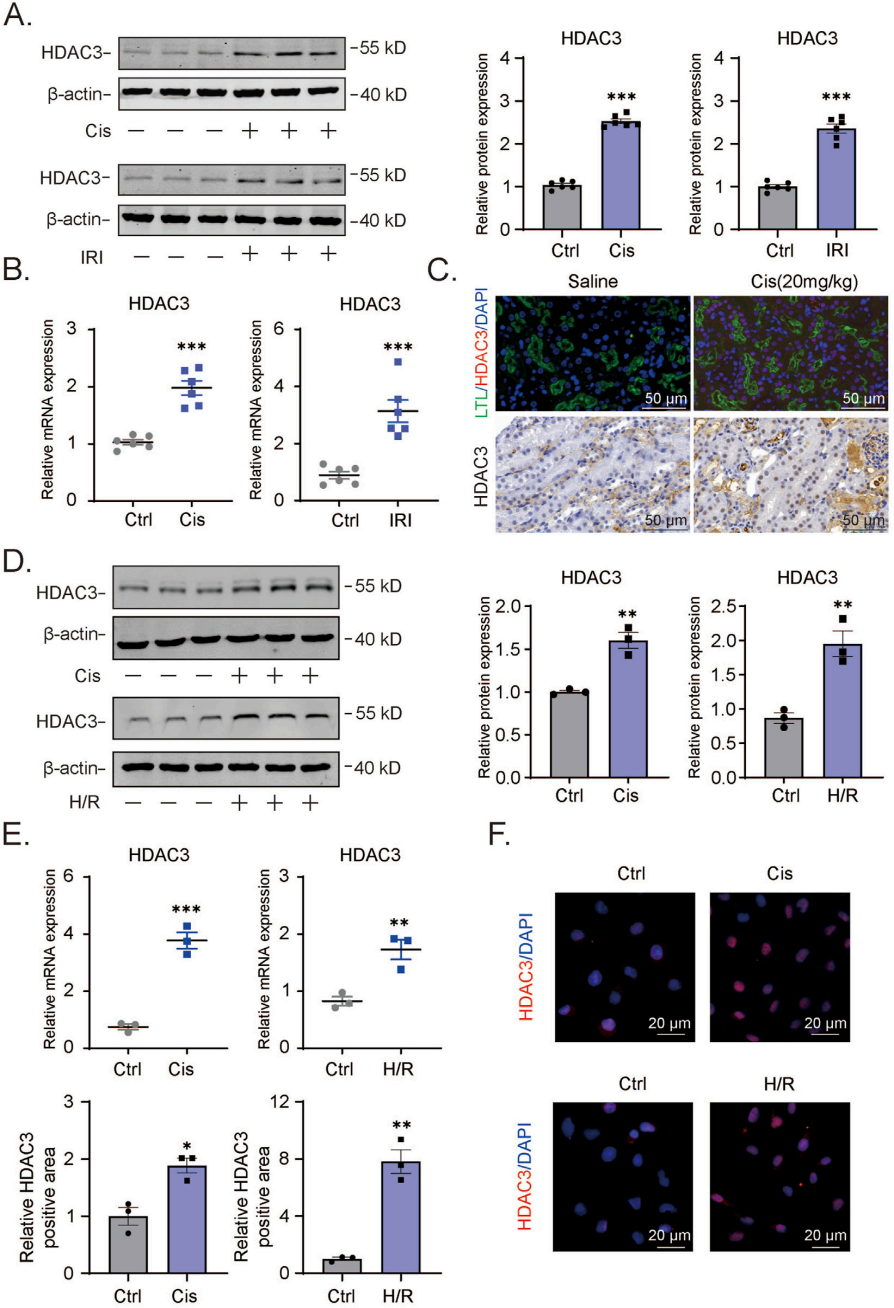
Increased HDAC3 expression in AKI mouse or cell model showcasing renal tubular epithelial cell injury **(A)**. The HDAC3 protein levels in kidney tissue of cisplatin-induced AKI mice were determined by Western blotting. **(B)**. The relative HDAC3 mRNA levels in kidney tissue of cisplatin-induced AKI mice were determined by qPCR. **(C)**. The HDAC3 expression and localization in kidney tissue of AKI mice were determined by immunofluorescency. **(D)**. The HDAC3 protein levels in HK2 cells with cisplatin-induced or H/R-induced AKI determined by Western blotting. **(E)**. The qPCR results of HDAC3 mRNA in cisplatin-induced or H/R-induced HK2 cells. **(F)**. Immunofluorescency of HDAC3 expression in cisplatin-induced or H/R-induced HK2 cells. Data are presented as the mean ± S.E.M. for at least 3–4 independent experiments or at least six mice. *P < 0.05; **P < 0.01; ***P < 0.001, relative to the control.

For the reasons disclosed in the Introduction, we undertook a comparative analysis of HDAC3 levels in control and kidney tissues from our AKI models based on cisplatin and I/R regimens, respectively. Relative to the control group tissues (saline injection and sham surgery), Western blotting and qPCR analyses detected significant increases in HDAC3 protein and mRNA levels associated with cisplatin and I/R (Figures 1A,B). Histological staining of kidney tissues with PAS confirmed the disruption of kidney architecture in the AKI models (Figure 1C bottom and data not shown). Further immunofluorescence staining against HDAC3 (red) reflected the increase in HDAC3 expression accompanying AKI with LTL (Leptospirillum lucidum) co-staining (green) showing that HDAC3 was the predominantly expressed in proximal tubule epithelium (Figure 1C top). We supplemented these findings by subjecting HK2 cells to cisplatin treatment to induce cell damage along with *in vitro* hypoxia-reoxygenation (H/R) to mimic the effects of the I/R model. Consistent with the *in vivo* AKI models, we observed significant increases in both HDAC3 mRNA and protein expression using Western blotting and qPCR, respectively (Figures 1D,E). Furthermore, immunofluorescence staining against HDAC3 was increased following either cisplatin or H/R treatments (Figure 1F).

### 3.2 RGFP966 reduces cisplatin-induced HK2 cell damage and production of inflammatory factors

**FIGURE 2:**
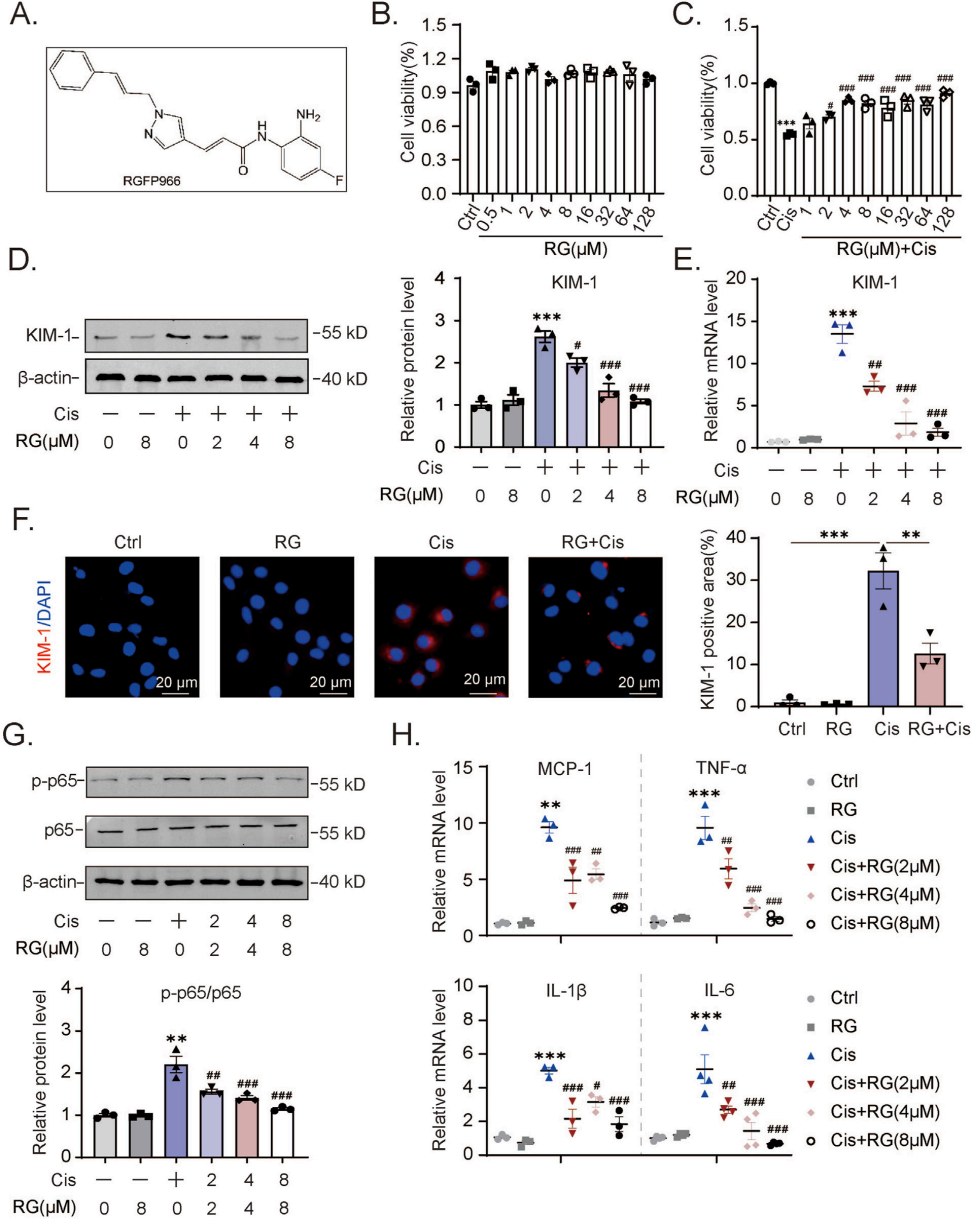
RG reduces cisplatin-induced damage and inflammation in HK2 cells **(A)**. The molecular structural formula of RG. **(B)**. The effect of different concentrations of RG on HK2 cell viability. **(C)**. RG restored the cell viability of cisplatin-treated HK2 cells. **(D)**. Western blotting analysis of the expression of KIM-1 in HK2 cells. **(E)**. QPCR results of KIM-1 expression in HK2 cells. **(F)**. Immunofluorescency results of KIM-1 expression in HK2 cells. **(G)**. Western blotting results of p-p65 expression in HK2 cells induced by cisplatin. **(H)**. QPCR analysis to determine the mRNA levels of MCP-1, TNF-α, IL-6 and IL-1β in HK2 cells. Data are presented as the mean ± S.E.M. for at least 3–4 independent experiments. Relative to the normal group: *P < 0.05, **P < 0.01, ***P < 0.001. Relative to the cisplatin group: #P < 0.05; ##P < 0.01; ###P < 0.001.

Given the consistent induction of HDAC3 in different AKI models, we argued that HDAC3 likely plays a key role in the degradation of renal tubular epithelium during AKI. The availability of RGFP966, a highly specific HDAC3 inhibitor (Figure 2A), provided the ready means to test our hypothesis. Accordingly, we tested whether RGFP966 offered protection against AKI. Importantly, the viability of HK2 cells as measured by CCK-8 assays was not compromised by RGFP966 treatment over a broad range of concentrations (0–128 μM, Figure 2B). Nonetheless, pretreating the cells with RGFP966 before the addition of cisplatin showed dose-dependent effects in mitigating cisplatin-induced cytotoxicity, with the protective activity plateauing at 4 µM RGFP966 (Figure 2C). These findings suggested that RGFP966 protects HK2 cells against cisplatin.

To corroborate this conclusion, we measured the levels of KIM-1 in Western blotting and qPCR assays, a marker commonly used to assess renal tubular injury. Cisplatin treatment of HK2 cells increased their expression of KIM-1 protein and mRNA while the pre-addition of RGFP966 dampened these increases (Figures 2D,E). Treatment with RGFP966 alone at the highest 8 µM concentration did not affect KIM-1 levels. Similar findings were evident in immunofluorescence analysis where RGFP966 markedly inhibited the increases in KIM-1 staining resulting from cisplatin treatment (Figure 2F). Further evidence showing that RGFP966 acts selectively to reverse the effects of cisplatin were obtained by examining expression changes in p65 protein activation. Cisplatin treatment of HK2 cells promoted increases in p65 activation (ratio of phosphorylated p65 to total p65) which was effectively reversed by RGFP966 (Figure 2G). Lastly, RGFP966 treatment, along with cisplatin, dramatically reduced the expression of inflammation-related genes, including monocyte chemoattractant protein (MCP)-1, tumor necrosis factor (TNF)-α, interleukin (IL)-6 and IL-1β. Collectively, these experiments reveal an additional aspect of RGFP966’s actions in cisplatin-treated HK2 cells, specifically its role in dampening the production of inflammatory response mediators.

### 3.3 RGFP966 reduces HK2 cell damage and inflammation factor production under H/R conditions

**FIGURE 3:**
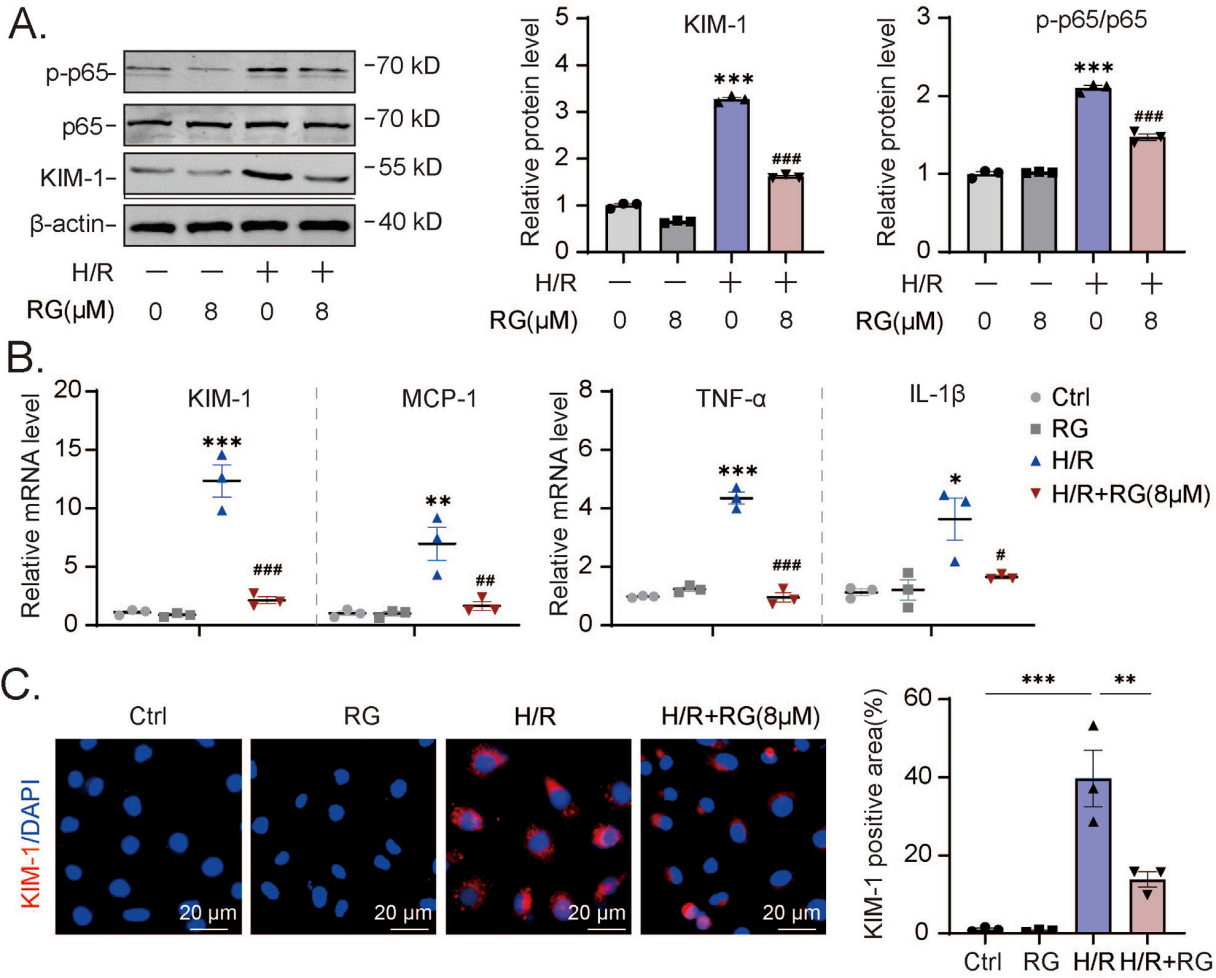
RG reduces H/R-induced cell damage and inflammation in HK2 cells **(A)**. Western blotting results of KIM-1 and p-p65 in H/R-induced HK2 cells treated with or without RG. **(B)**. The mRNA expression of KIM-1, MCP-1, TNF-α, IL-6 and IL-1β in **(A)**. **(C)**. The detection of the KIM-1 expression in H/R-induced HK2 cells treated with or without RG by immunofluorescency analysis. Data are presented as the mean ± S.E.M. of at least three independent experiments. Relative to the normal group: *P < 0.05, **P < 0.01, ***P < 0.001. Data relative to the H/R-induced group: #P < 0.05; ##P < 0.01; ###P < 0.001.

To expand the scope of our findings with cisplatin, we extended the HK2 cell experiments by employing hypoxia/reoxygenation (H/R) conditions as an alternative method to induce AKI effects. First, using Western blotting we observed that H/R induced KIM-1 expression as well as p65 activation could be alleviated, at least partially by RGFP966 (Figure 3A). Furthermore, the results of qPCR assays confirmed these effects on KIM-1 transcripts as well as showing that RGFP966 inhibited increases in production of MCP-1, TNF-α, IL-6, and IL-1β elicited by H/R (Figure 3B). Consistently, immunofluorescence assays indicated that the increase in KIM-1 staining was dramatically decreased in H/R-treated HK2 cells with RGFP966 pretreatment (Figure 3C). Thus, RGFP966 also protects HK-2 cells against the effects of H/R *in vitro*.

### 3.4 RGFP966 alleviates inflammation in cisplatin-induced AKI mice

**FIGURE 4:**
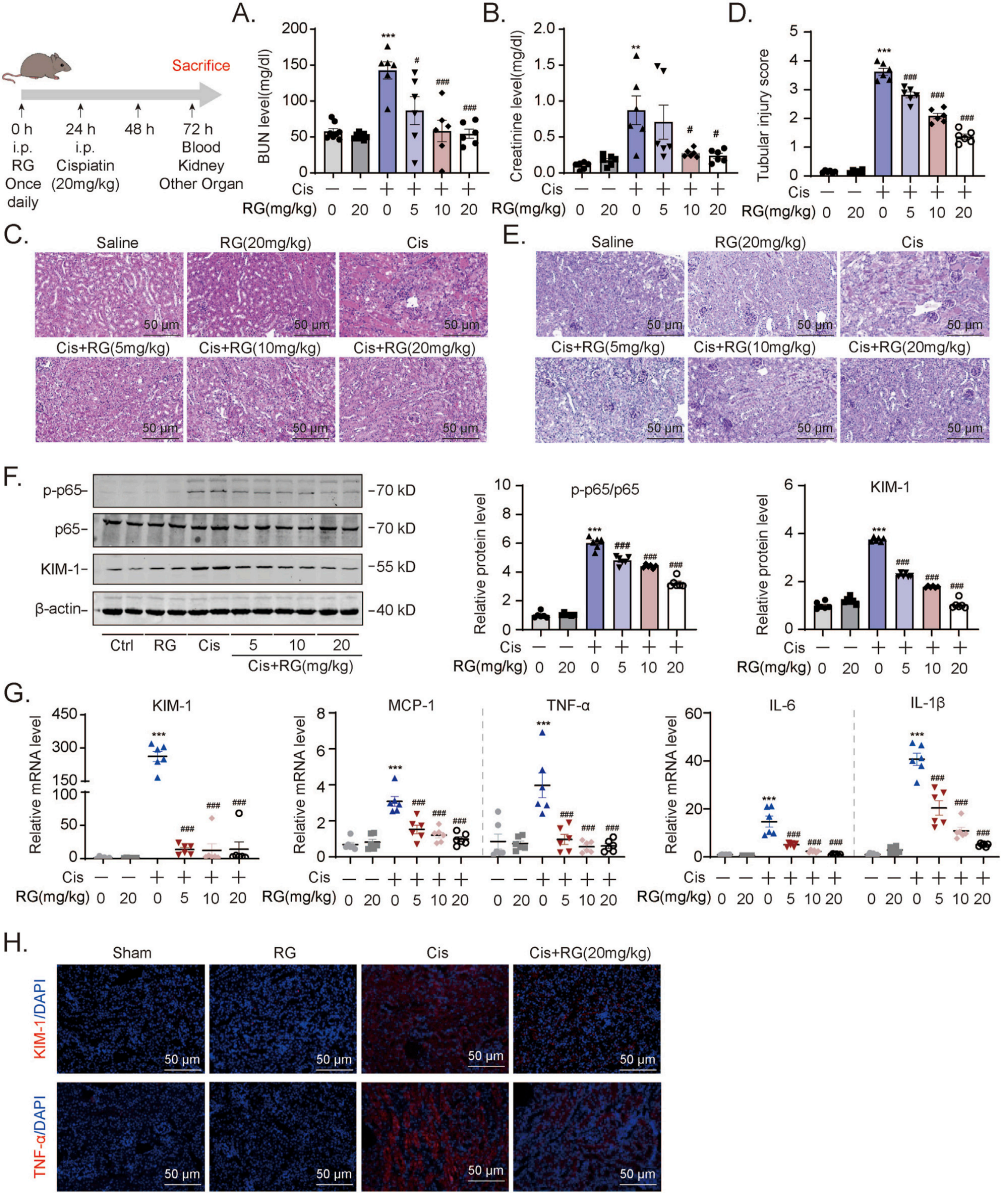
RG alleviates inflammation in cisplatin-induced AKI mice **(A)** and **(B)**. BUN **(A)** and Creatinine **(B)** assays for renal functions of AKI mouse model. **(C-E)**. The HE **(C)** and PAS staining **(D and E)** showing the effect of RG on the tubular dilatation and cast formation after cisplatin-induced nephropathy. **(F)**. Western blotting results of the expression of KIM-1 and p-p65. **(G)**. QPCR results showing mRNA expression of KIM-1, MCP-1, TNF-α, IL-1β and IL-6 to determine the inflammation. **(H)**. Immunofluorescency results in paraffin section revealing the effect of RG on the expression of KIM-1 and TNF-α in kidney injury. Data are presented as the mean ± S.E.M. of at least six independent mice. Data relative to the saline group: *P < 0.05, **P < 0.01, ***P < 0.001. Data relative to the cisplatin group: #P < 0.05; ##P < 0.01; ###P < 0.001.

Building on our *in vitro* findings, we next investigated whether RGFP966 could effectively prevent AKI-associated pathogenic effects *in vivo*. We first pretreated mice with RGFP966 (5, 10 and 20 mg/kg) 12 h prior to cisplatin, repeating administration once a day for 3 days. Cisplatin caused significant increases in blood urea nitrogen and creatinine levels while RGFP966 pretreatment resulted in dose-dependent decreases in their respective levels (Figures 4A,B). Parallel histological assessment of renal tissues using hematoxylin-eosin and PAS staining of renal tissues confirmed that RGFP966 attenuated cisplatin-induced tubular dilatation and cast formation (Figures 4C,E). In support of these findings, RGFP966 dampened p65 activation and KIM-1 protein and mRNA expression increases resulting from cisplatin treatment (Figures 4F,G). Moreover, RGFP966 significantly inhibited the transactivation of inflammatory mediators including MCP-1, TNF-α, IL-6 and IL-1β (Figure 4G) with immunofluorescence staining confirming lowered levels of TNF-α and KIM-1 in renal tissues from RGFP966/cisplatin treated mice (Figure 4H).

### 3.5 RGFP966 attenuates acute renal damage and inflammation caused by I/R

**FIGURE 5:**
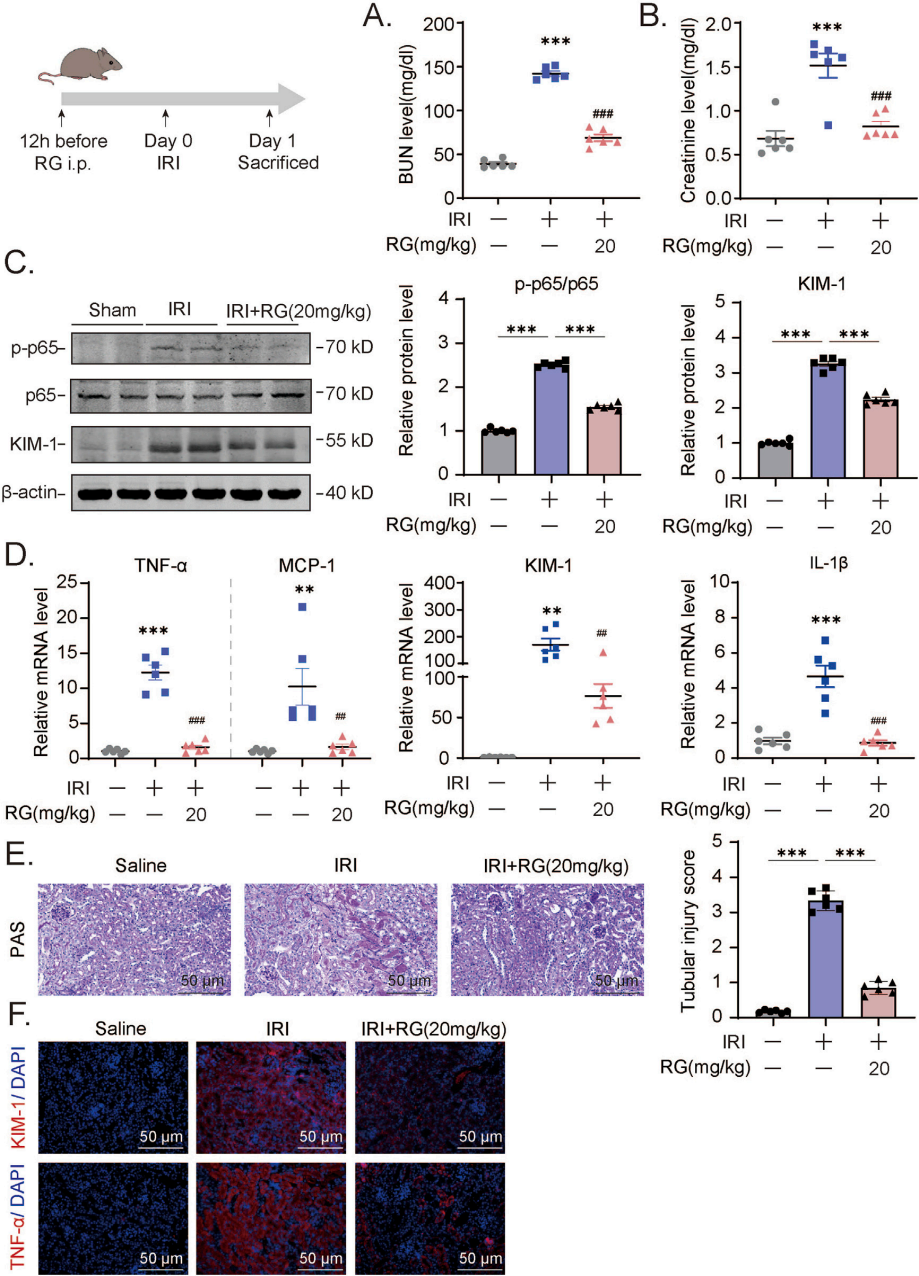
RG attenuates the acute renal damage and inflammation induced by IRI *in vivo* **(A-B)**. The detection of BUN **(A)** and creatinine **(B)** levels in IRI mouse model. **(C)**. Western blotting results of the expression of KIM-1 and p-p65 in the kidney tissue of IRI mouse. **(D)**. QPCR results of mRNA expression of TNF-α, MCP-1, KIM-1, IL-1β to determine the inflammation. **(E)**. PAS staining and corresponding scoring analysis. **(F)**. Immunofluorescency analysis of paraffin section for KIM-1 and TNF-α expression. Data are presented as the mean ± S.E.M. of at least six independent mice. Data relative to the saline group: *P < 0.05, **P < 0.01, ***P < 0.001. Data relative to the IRI-induced group: #P < 0.05; ##P < 0.01; ###P < 0.001.

We further tested whether RGFP966 would also influence AKI-related renal damage in the I/R mouse model. Indeed, RGFP966 pretreatment similarly protected against the effects of I/R as shown by its prevention of elevations in blood urea nitrogen and creatinine levels (Figures 5A,B). Moreover, RGFP966 also reversed, at least partially, the activation of p65 and increases in KIM-1 protein and mRNA levels (Figures 5C,D) with more marked effects on the inhibition of TNF-α, MCP-1, and IL-1β transactivation by I/R treatment (Figure 5D). Histopathological assessment using PAS staining also confirmed that RGFP966 pretreatment rescued tubular dilatation and necrosis in the renal tissues of IRI-treated mice (Figure 5E) while the results of immunofluorescence staining confirmed the reductions in KIM-1 and TNF-α expression at the protein level (Figure 5F). Thus, RGFP966 exerts protective effects against renal dysfunction in different *vivo* models of AKI.

### 3.6 RGFP966 alleviates cisplatin-induced programmed necrosis of HK2 cells

**FIGURE 6:**
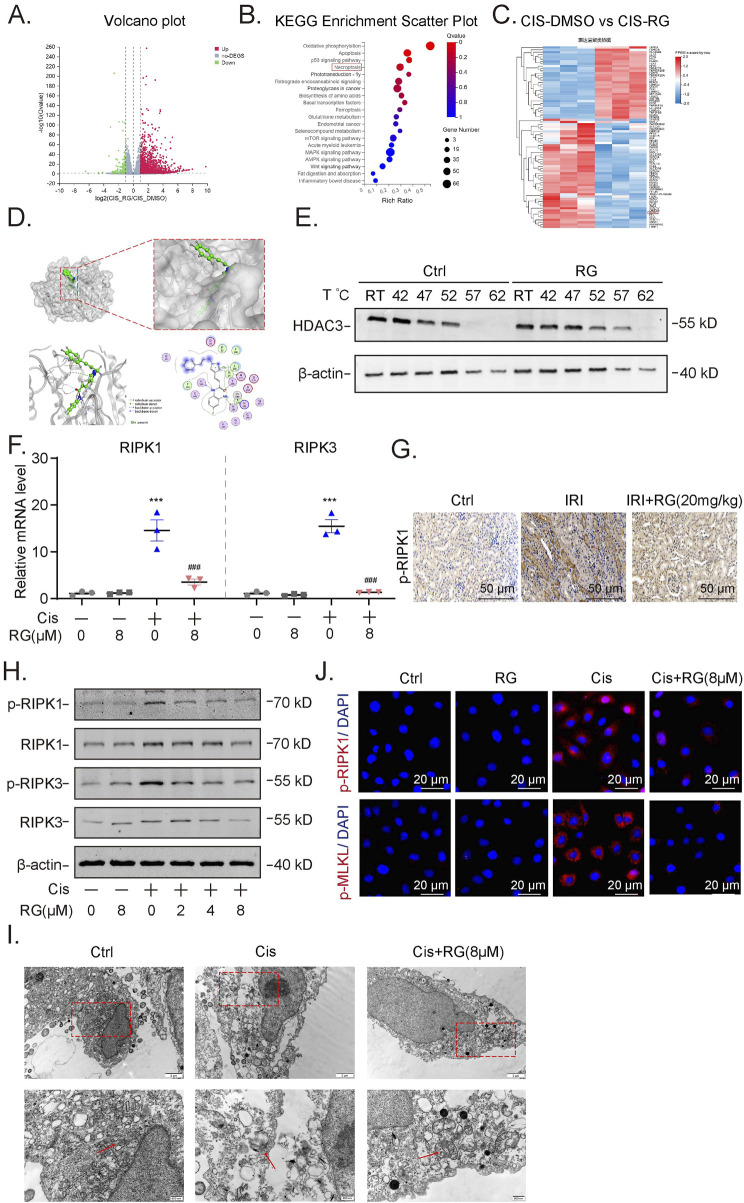
RG attenuates cisplatin-induced programmed necrosis of HK2 cells **(A)**. Volcano Maps of the RNA-seq showing differential expressed genes. **(B)**. KEGG Enrichment Scattter of RNA-seq. **(C)**. Heat map showing upregulated genes including RIPK1. **(D)**. Molecular docking analysis revealed the physical binding of RG to HDAC3. **(E)**. Western blotting results showing the stability of HDAC3 in HK2 cells without and with RG treatment by CETSA assays. **(F)**. QPCR detection the mRNA expression of RIPK1 and RIPK3. **(G)**. Immunohistochemistry analysis of p-RIPK1 in the kidney of I/R-induced AKI mice. **(H)**. Western blotting results showing the expression of p-RIPK1/RIPK1 and p-RIPK3/RIPK3. **(I)**. Representative electron micrographs showing the necrotic HK2 cells. **(J)**. Immunofluorescency results of p-RIPK1 and p-MLKL in cisplatin-treated HK2 cells. Data are presented as the mean ± S.E.M. of at least three independent experiments or at least six mice. *P < 0.05; **P < 0.01; ***P < 0.001, relative to the NC group. #P < 0.05; ##P < 0.01; ###P < 0.001 relative to the treated group.

To glean clues as to how RGFP966 exerts the aforementioned effects, we undertook RNA-seq-based analysis of HK2 cells treated without and with RGFP966 in conjunction with cisplatin treatment. Using the significantly differentially expressed genes to conduct KEGG pathway enrichment analysis, we found that necroptosis (programmed necrosis pathway) ranked highly among the enriched pathways (Figures 6A,B). Moreover, hierarchical cluster of the altered genes showed many were involved in classical RIPK1 signaling (Figure 6C) with subsequent qPCR analysis showing that the upregulation of both RIPK1 and RIPK3 by cisplatin was countered by RGFP966 pretreatment (Figure 6F). Further Western blotting analysis showed that RGFP966 reduced the expression and activation status (phosphorylation at Ser166; p-RIPK1) of RIPK1 and RIPK3 in cisplatin-induced HK2 cells (Figure 6H). Consistently, immunofluorescence staining confirmed the effects of RGFP966 in preventing cisplatin-induced increases in p-RIPK1 expression along with phosphorylation-induced activation of the upstream the necroptotic activator MLKL (Figure 6J). At the ultrastructural level, Transmission electron microscopy (TEM) showed cisplatin induced characteristic features of necroptosis in HK2 cells, such as nuclear membrane crumpling, widening, cell membrane dehiscence, mitochondrial swelling and deformation while pretreatment with RGFP966 alleviated many of these features (Figure 6I). Lastly, immunohistochemistry of renal tissues from the I/R mouse model showed RGFP966 pretreatment reduced p-RIPK1 staining (Figure 6G), implying that RGFP966 similarly prevents against necroptosis in renal tubules *in vivo*.

### 3.7 The protective effects of RGFP966 in AKI result from inhibition of RIPK1-mediated programmed necrosis

**FIGURE 7:**
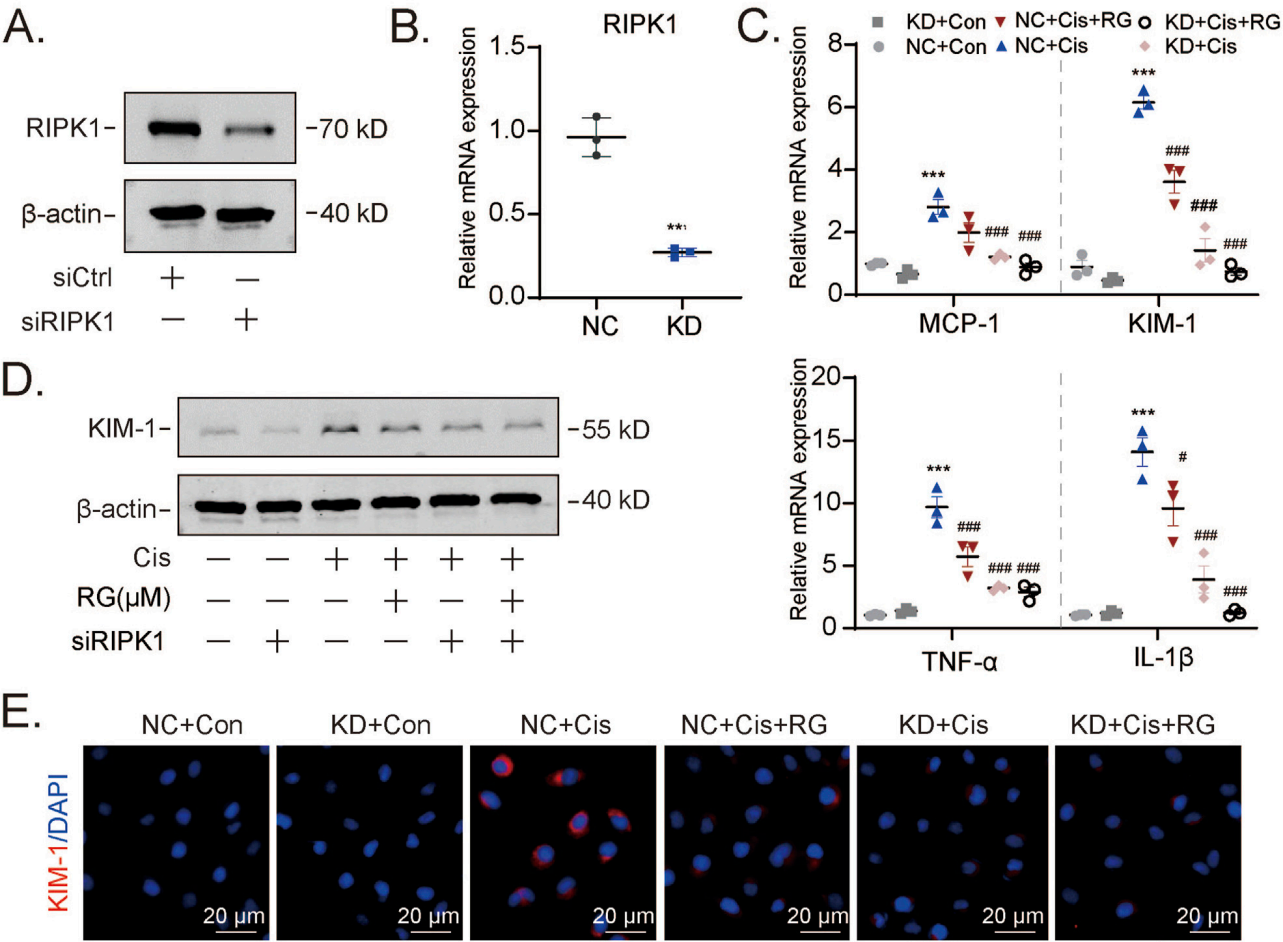
RG exerts a protective effect by regulating RIPK1-mediated programmed necrosis **(A and B)**. Western blotting **(A)** and qPCR **(B)** results showing the knockdown efficiency of RIPK1 at protein and mRNA levels. **(C-E)**. Western blotting, qPCR and immunofluorescency analysis of molecules related to kidney injury including KIM-1, MCP-1, TNF-α and IL-1β. Data are presented as the mean ± S.E.M. of at least three independent experiments ±S.E.M. Relative to the normal group: *P < 0.05, **P < 0.01, ***P < 0.001. Data relative to the cisplatin group: #P < 0.05; ##P < 0.01; ###P < 0.001.

Our preceding data associated the protective effects of RGFP966 with necroptosis, although we could not exclude the significant involvement of other forms of programmed cell death. To strengthen this connection, we used small interfering RNAs (siRNAs) to knockdown RIPK1 in HK2 cells. Effective RIPK1 knockdown at both mRNA and protein levels was first confirmed by qPCR and Western blotting (Figures 7A,B). Thereafter, we treated control and RIPK1 knockdown HK2 cells with cisplatin alone or combined with RGFP966 pretreatment. Instructively, RIPK1 depletion abolished the impact of RGFP966 as shown by prevention of mRNA and protein level increases of KIM-1 (Figures 7C,D), along with proinflammatory marker mRNA expression (Figure 7E) and immunofluorescence staining against KIM-1 (Figure 7E). Collectively, it can be concluded that RGFP966 reduces acute kidney damage *via* effects on RIPK1-mediated programmed necrosis.

### 3.8 Multiorgan safety assessment of RGFP966 in mice

**FIGURE 8:**
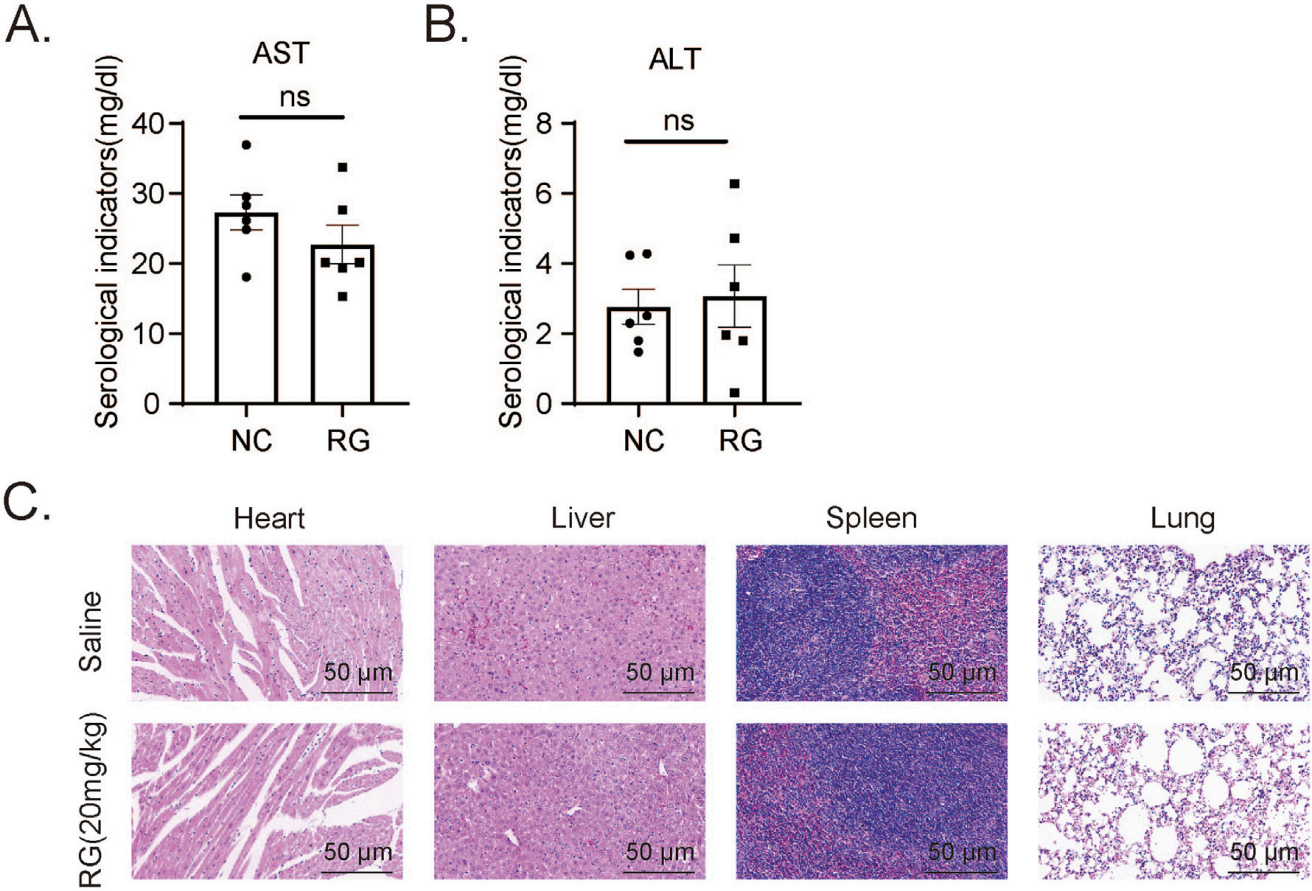
Effects of RG on the tissues including heart, liver, spleen and lungs of normal mice **(A and B)**. The AST **(A)** and ALT **(B)** levels in serum of normal mice. **(C)** H&E staining results of different tissues. Data are presented as the mean ± SEM for 6–8 independent experiments.

The efficacy and safety of any therapeutic agent necessitate a comprehensive evaluation of its dosing parameters, pharmacodynamic properties, and potential off-target effects, with particular attention to markers such as liver function due to its role in drug metabolism. After administration of RGFP966 (20 mg/kg) or saline (control) to normal mice we observed no significant differences in blood liver function markers (ALT and AST) (Figures 8A,B). H&E staining performed to assess the impact of RGFP966 on other organs, including the liver, heart, spleen and lung showed remarkably similar histological appearance (Figure 8C). Thus preliminary evidence suggests RGFP966 is safe in mice at the 20 mg/kg dose required to maximally reduce AKI pathologies in our models.

## 4 Discussion

In the context of both *in vitro* and *in vivo* AKI model systems, our study provides two major but interrelated conclusions. First, that HDAC3 is crucial for the development of AKI and second that targeting HDAC3 with a specific inhibitor, RGFP966, is sufficient to prevent related renal pathologies.

The *in vivo* studies indicated that HDAC3 exacerbates kidney damage in mice with cisplatin-induced AKI. Additionally, the protective effect of the HDAC3 inhibitor RGFP966 was established in both cell model and mouse model. Our investigations demonstrated that RGFP966 acts against cisplatin and H/R-induced AKI in HK2 cell model and C57BL/6 mouse model. Moreover, the RNA-seq results also showed that RGFP966 is beneficial in attenuating AKI, both *in vitro* and *in vivo*, by preventing inflammation and programmed necrosis. Additionally, RGFP966 was proven to be an effective modulator of RIPK1-mediated programmed necrosis, thereby ameliorating cisplatin-induced AKI. Altogether, these findings implicate that RGFP966 may be a potential candidate for treating AKI.

First, in this study, HDAC3 was found to be significantly upregulated in mice with cisplatin-induced AKI, especially in proximal renal tubular epithelial cells. This finding was confirmed in the *in vitro* model of cisplatin- and H/R-induced AKI. Histone deacetylases and acetyltransferases are primarily responsible for regulating histone modifications and are vital for cell survival, homeostasis, cell proliferation, and gene expression (Sarkar et al., 2020; Amin et al., 2017). Furthermore, Changlong An, *et al.* reported that the absence of HDAC3 in myeloid cells reduces the accumulation of bone marrow-derived fibroblasts and the infiltration of macrophages. This, in turn, alleviates angiotensin II-induced kidney damage and fibrosis, thereby providing renal protection (An et al., 2023). Inhibition of HDAC enzymes as a therapeutic approach to diseases has attracted immense attention in recent years. Designing small-molecule HDAC inhibitors for numerous disorders, including cancers, is emerging as an important area of research (Li et al., 2021). These inhibitors include that of HDAC class I (1, 2, 3, and 8), HDAC class IIa (4, 5, 7, and 9) and HDAC class IIb (6, 10) (de la Fuente Revenga et al., 2018; Huang et al., 2022; J. Yang et al., 2021; W. Yang et al., 2021; Chen et al., 2021). Although the role of HDAC3 selective inhibitors in treating renal fibrosis has been explored and appears to be a viable option, their potential role in AKI and the underlying mechanism are yet to be determined (Sato and Yanagita, 2018).

Second, in animal models with AKI induced by cisplatin and I/R, RGFP966 decreased the inflammation dramatically. These findings were further supported by the *in vitro* experiments. AKI frequently results in inflammation due to triggers such as ischemia and hypoxia, infection and nephrotoxic medications (Gao et al., 2018). Severe or persistent AKI often leads to chronic inflammation, fibrosis in the kidneys, tubular degeneration, and finally chronic kidney disease (CKD) (Ferenbach and Bonventre, 2015; Q. Yang et al., 2021). Therefore, treating AKI and preventing its transition to CKD *via* anti-inflammatory therapy is a promising strategy to reduce renal damage. By preventing IGFBP7/IGF1-mediated programmed cell death and inflammation, we previously demonstrated that gibberellin combined with saponin may prevent AKI (Gao et al., 2016). Cpd-6c, a rutaecarpine derivative that targets PDE4B, is an important regulator driving irritation in cisplatin-induced kidney disease and has been reported to alleviate AKI (Liu et al., 2020). Protocatechuic aldehyde has been shown to reduce AKI by inhibiting inflammation and oxidative stress in animal models (Bertheloot et al., 2021). This finding confirmed the significance of inflammation in mediating AKI. Here, our findings indicated that RGFP966 had a beneficial impact on inflammation both *in vitro* and *in vivo* by inhibiting the expression of numerous inflammatory factors, such as MCP-1. Additionally, NF-κB p65 activation, a well-known mechanism in renal inflammation, is drastically inhibited by RGFP966.

Third, the present study suggested that RGFP966 prevents programmed necrosis both *in vivo* and *in vitro.* Necroptosis, also known as programmed necrosis, is a regulated inflammatory cell death mechanism mediated by the activation of RIPK1, RIPK3 and downstream MLKL. It is noted that the necroptosis differs from apoptosis morphologically, in that the cell swells, cell membrane tears, and the cytoplasmic contents are released (Lawlor et al., 2015). The usual triggers for necroptosis are external stimulations. When a ligand such as TNF-α binds to the death receptor on the cell membrane, it results in cell death. The receptors TNFR1, Fas/CD95, DR4/TRAIL-R1, and DR5/TRAIL-R2 belong to the TNF-α superfamily. These receptors, in their active form, bind to adaptor proteins TRADD and TRAF2, thereby delaying the initiation of RIPK1 (Ketelut-Carneiro and Fitzgerald, 2022; Frank and Vince, 2019b). Pathogen activation of PRRs, such as toll-like receptors, which in turn activate the interacting, RIPK1-independent, but RIPK3-activating articulators TRIF and ZBP-1 are additional causes of necroptotic apoptosis (Lawlor et al., 2015; Xu et al., 2021). The fact that RIPK1 is tightly controlled and involved in the initiation of NF-κB p65 signaling; moreover, the complexes of survival, apoptosis, or necrotic apoptosis should be recorded (Lawlor et al., 2015; Wang et al., 2019a). Our team has been investigating RIPK1/RIPK3 and its role in necrotic apoptosis for years, and our previous study showed that the RIPK1 antagonist Cpd-71 prevents cisplatin-induced renal insufficiency by reducing necroptosis and inflammation (Wang et al., 2019b).

The findings of the present study implied that RGFP966 has renal protective effects and its mechanism was further examined, and RNA-seq analysis showed that the necrosis and inflammatory pathways ranked high in the KEGG pathway enrichment analysis. Therefore, it is hypothesized that RGFP966 may exert its protective effect by attenuating programmed necrosis. The results of Western blotting and real-time qPCR of RIPK1 and RIPK3 confirmed this hypothesis. In addition, RIPKI-knockdown experiments demonstrated that RGFP966 attenuated AKI by regulating RIPK1-mediated programmed necrosis.

Overall, RGFP966 attenuated cisplatin and IRI-induced AKI *in vivo*, necroptosis induced by cisplatin and H/R *in vitro*, and suppressed inflammation, thereby alleviating HK2 injury. Moreover, the administration of RGFP966 had no adverse effects on the heart, liver, spleen or lung tissues of normal C57BL/6 mice. These findings suggest that RGFP966 is a potentially safe clinical candidate for treating AKI.

Lastly, while our findings are promising, our study has some limitations which need to be acknowledged. Our results are entirely based on experimental models requiring further confirmation in clinical specimens. Moreover, although RGFP966 appears safe in mice in the short-term, longer-term experiments and pharmacological studies are required to bridge the gap to human subjects. While our study primarily focuses on the role of HDAC3 in necroptosis and apoptosis, we recognize that HDAC3 may also regulate alternative pathways that contribute to kidney injury. For example, IL-4-induced activation of STAT6 facilitates transcriptional repression by recruiting the NCoR-HDAC3 complex to specific target genes, representing a key molecular mechanism underlying STAT6-dependent gene suppression. In addition, Baihai Jiao, *et al.* reported that inhibition of STAT6 reduced M2 macrophage polarization and attenuated renal fibrosis (Czimmerer et al., 2018; Jiao et al., 2021).Moreover, the precise mechanisms linking HDAC3 with effects on RIPK1 expression and necroptosis need to be explored in better detail to determine how to best suppress this pathway in the clinical context.

## Data Availability

The data presented in the study are deposited in the NCBI repository, accession number PRJNA1249933.

## Abbreviations

Cr, Creatinine; RG: RGFP966; IF, Immunofluorescence; HK2, Human kidney tubular epithelial cells; H/R, Hypoxia/reoxygenation; IRI, Ischemia Reperfusion Injury; CKD, Chronic Kidney Disease; I/R, Ischemia/reperfusion; TNF-α, Tumor Necrosis Factor-α; CETSA, Cellular thermal shift assay; Cis, Cisplatin; RNA-seq, RNA Sequencing; RIPK1, Receptor-Interacting Protein Kinase 1; RIPK3, Receptor-Interacting Protein Kinase 3; PAS, Periodic acid schiff; H&E, Hematoxylin and eosin; BUN, Blood urea nitrogen; HDAC3i, Histone deacetylase 3 inhibitor; AST, Aspartate aminotransferase; ALT, Alanine aminotransferase; AKI, Acute kidney injury.
