# Supplementary material for: HDAC3 inhibition mitigates acute kidney injury by alleviating RIPK1-mediated programmed necrosis
Source: Front Pharmacol. 2025 Apr 25;16:1546950. doi: 10.3389/fphar.2025.1546950 (PMC12061726; doi:10.3389/fphar.2025.1546950)
Supplement: Supplementary file 1 [file Table1.doc]

**Supplement Table 1. Primers and RNA sequences were used in this study.**

1. Primers for real-time PCR (human)

| Terms Forward primer (5'-3') Reverse primer (5'-3') |
| --- |

KIM-1 CTGCAGGGAGCAATAAGGAG TCCAAAGGCCATCTGAAGAC

TNF-α CCCAGGGACCTCTCTCTAATCA GCTACAGGCTTGTCACTCGG

IL-6 CGGGAACGAAAGAGAAGCTCTA GAGCAG CCCCAGGGAGAA

IL-1β ACTACAGCAAGGGCTTCAGG CATATCCTGTCCCTGGAGGT

MCP-1 AGCAGCAAGTGTCCCAAAGA GGTGGTCCATGGAATCCTGA

β-actin CGCCGCCAGCTCACCATG CACGATGGAGGGGAAGACGG

HDAC3 GGCTTCTGCTATGTCAACGA CCTTTTAAACCTCCCCAGCA

RIPK1 AGACTAGGTGGCAGGAAAGAA ACACCTCATCTAAAGGCTGG

RIPK3 GCTGTCTCCACGGTAAAGG AGCCACTCAGAAACCATGAC

1. Primers for real-time PCR (mouse)

| Terms Forward primer (5'-3') Reverse primer (5'-3') |
| --- |

KIM-1 CAGGGAAGCCGCAGAAAA GAGACACGGAAGGCAACCAC

TNF-α CATCTTCTCAAAATTCGAGTGACAA TGGGAGTAGACAAGGTACAACCC

IL-6 GAGGATACCACTCCCAACAGACC AAGTGCATCATCGTTGTTCATACA

IL-1β GCTTCAGGCAGGCAGTAT ACAAACCGCTTTTCCATCT

MCP-1 CTTCTGGGCCTGCTGTTCA CCAGCCTACTCATTGGGATCA

β-actin CATTGCTGACAGGATGCAGAA ATGGTGCTAGGAGCCAGAGC

HDAC3 CCCCACCAATATGCA GGGTT CAGAAGCCAGAGGCCTCAAA
